# Special Issue: Honey Bee Viruses

**DOI:** 10.3390/v7102885

**Published:** 2015-10-26

**Authors:** Sebastian Gisder, Elke Genersch

**Affiliations:** Institute for Bee Research, Department of Molecular Microbiology and Bee Diseases, Friedrich-Engels-Str. 32, 16540 Hohen Neuendorf, Germany; gisderse@hu-berlin.de

**Keywords:** Lake Sinai Viruses (LSV), bee macula-like virus (BeeMLV), *Apis mellifera* filamentous virus (AmFV), chronic bee paralysis virus (CBPV), Israeli acute paralysis virus (IAPV), RNAi approach, honey bee viruses, *Apis mellifera*, *Bombus terrestris*

## Abstract

Pollination of flowering plants is an important ecosystem service provided by wild insect pollinators and managed honey bees. Hence, losses and declines of pollinating insect species threaten human food security and are of major concern not only for apiculture or agriculture but for human society in general. Honey bee colony losses and bumblebee declines have attracted intensive research interest over the last decade and although the problem is far from being solved we now know that viruses are among the key players of many of these bee losses and bumblebee declines. With this special issue on bee viruses we, therefore, aimed to collect high quality original papers reflecting the current state of bee virus research. To this end, we focused on newly discovered viruses (Lake Sinai viruses, bee macula-like virus), or a so far neglected virus species (*Apis mellifera* filamentous virus), and cutting edge technologies (mass spectrometry, RNAi approach) applied in the field.

## 1. Introduction

Although the number of managed honey bee colonies has increased globally by more than 45% over the last 60 years [1,2,3], there is growing concern regarding the health status of the existing honey bee colonies. Over the last 15 years, dramatic colony losses over winter have been reported frequently from different regions all over the world [4,5]. Many of these losses eluded easy explanations and, hence, evoked intensive multi-disciplinary research into this phenomenon. By now it is consensus (i) that such losses are in most cases multifactorial and can hardly be attributed to a single cause and (ii) that the combination of factors resulting in colony losses differs from time to time and region to region. However, most often virus infections are among the key players in colony losses and, hence, viruses are rightly considered a major threat to the health of honey bees, both at individual and at colony level.

In reaction to this insight, about ten years ago virologist Michel Aubert started a European initiative called BRAVE (Bee Research and Virology in Europe) to identify research gaps and research needs in the field of bee virology. The aim was to collect the knowledge on honey bee viruses available at that time in order to create a basis for state-of-the-art research because “honey bees must be protected against virus diseases” [6]. This effort was seminal and, indeed, since then the knowledge on bee viruses and bee virology has improved considerably. Therefore, it was the right time now to publish a special issue on honey bee viruses giving a snapshot of the most recent findings and novel developments in the field.

## 2. Honey Bee Viruses

Two original papers in this special issue deal with a rather neglected but widely distributed virus, *Apis mellifera* filamentous virus (AmFV). Interestingly, amongst the 23 honey bee viruses described to date [7,8,9,10], AmFV is the only honey bee virus which has a double stranded DNA-genome [11]. Originally, the disease associated with AmFV-infection was thought to be caused by *Rickettsia* when it was first described in the early sixties of the last century [12,13] and, hence, it was named rickettsiosis. The small particles present in the milky white hemolymph of infected honey bees were classified as *Rickettsia melolontha* [13]. It took more than 10 years before it became evident in electron micrographs of thin sections of honey bees, which showed the symptoms of the rickettsiosis, that these small particles actually were nucleocapsids of filamentous virions [14]. AmFV was described as a pathogen with low virulence which normally does not cause any overt disease symptoms. Infected bees may show symptoms of weakness but individual life span seems not to be shortened [14]. According to historical field data, AmFV infections could only be detected in *Nosema apis* infected bees, suggesting that *N. apis* infection is a prerequisite for an AmFV infection to become established [15]. Infections with AmFV have not been correlated to colony losses so far [14,16]. However, AmFV has recently been shown to be prevalent in many solitary bee species [17]. To understand virus-host-interactions of AmFV and bees on the molecular level it is important to have access to the AmFV genome sequence. In the study “The *Apis mellifera* Filamentous Virus Genome” by Gauthier and co-workers in this issue [18], the authors determined the genomic sequence of AmFV thus providing so far unavailable information on virus genome characteristics and virus classification. The double stranded DNA-genome has 498.500 nucleotides and represents the biggest known honey bee virus genome. When two isolates from different continents were compared, the genome was found to be highly conserved. One of the key findings of the study was the similarity of thirteen open reading frames (ORF) with typical baculovirus domains. *In silico* analysis of the AmFV genome also revealed many so far unknown ORFs without data base matches. This result underlines the uniqueness of this virus amongst all other honey bee viruses and emphasizes the importance of molecular data on honey bee viruses in order to further our understanding of their evolution and complex relationship with their host(s).

AmFV is supposed to be of low virulence and establishment of infection was thought to be dependent on pre-existing *N. apis* infections [15] although no controlled studies or experimental data were available for this notion. Therefore, a study like the one presented by Hartmann and co-workers on the “Dynamics of *Apis mellifera* Filamentous Virus (AmFV) Infections in Honey Bees and Relationships with Other Parasites” [19] was overdue. The authors analyzed the prevalence of AmFV in Swiss honey bee colonies and the relationship between AmFV and other common honey bee pathogens in honey bee colonies from Switzerland, France, and Sweden. Interestingly, the majority of the bees (up to 75%) developed detectable levels of AmFV infection during the first week post emergence and virus titers remained constant over time suggesting chronic infections. However, no disease symptoms could be observed at individual bee or colony level, hence, AmFV is rather causing covert infections. Analyzing the relation between AmFV and other bee pathogens revealed a positive correlation between AmFV infections and infections with DWV (deformed wing virus), SBV (sacbrood virus), and BQCV (black queen cell virus), as well as a potentially negative association between infections with AmFV and trypanosomes. No association could be observed between AmFV and *Varroa destructor* suggesting that *V. destructor* does not act as a vector for AmFV. Contradictory to historical data [15], there was also no association between the detection of AmFV and *Nosema* spp., hence, preexisting *Nosema* spp. infections do not seem to be a prerequisite for AmFV infections to become established. Taken together, the study by Hartmann and co-workers does not substantiate a major role for AmFV infections in honey bee colony collapse, although it cannot be ruled out that a general weakening of individual bees through AmFV infections might make them more susceptible to other pathogens.

The study “Genome Characterization, Prevalence and Distribution of a Macula-Like Virus from *Apis mellifera* and *Varroa destructor*” by de Miranda and co-workers [20] is focused on a novel honey bee virus, tentatively named bee macula-like virus (BeeMLV), which was originally thought to be a plant virus contamination in a DWV sequencing approach from 2001 (21). Genomic analysis and characterization placed this virus into the family *Tymoviridae* in the order *Tymovirales*. The *Tymoviridae* comprise three genera (*Maculavirus*, *Marafivirus*, and *Tymovirus*) and BeeMLV is closest to the genus *Maculavirus*. BeeMLV has a polyadenylated RNA genome with about 6500 nucleotides. This entirely new virus was found replicating in honey bees and presumably also in *V. destructor*. The high titers of BeeMLV in infected adult and pupae samples indicate that honey bees are definitely a host but *V. destructor* may be a biological vector for this virus rather than a true host. Further investigations are needed to unravel whether BeeMLV is an endemic or an incidental virus, to identify the host spectrum of this virus, and to analyze its transmission routes as well as pathology in and virulence for bees and bee colonies.

Most recently, the Lake Sinai virus (LSV) group has been discovered as very prevalent and widely distributed bee infecting viruses [9]. Phylogenetic studies based on protein sequences of the RNA dependent RNA polymerase (RdRP) and the capsid proteins of LSVs both placed this unclassified virus group in vicinity to chronic bee paralysis virus (CBPV), although LSVs form their own monophyletic *Sinaivirus* clade [22]. LSV was present in *V. destructor*, albeit viral replication could not be detected [22]. In their study “Honey Bee Infecting Lake Sinai Viruses”, Daughenbaugh and co-workers [23] analyzed the currently circulating LSVs in terms of sequence variation, host range, prevalence, and relation to colony health. The authors identified two more LSVs, LSV6 and LSV7, in honey bee samples and confirmed the presence of LSV1 and LSV2 in *V. destructor* [22]. They also confirmed that LSVs form a separate monophyletic clade related to but distinct from CBPV. Monitoring six bee colonies for three months revealed a statistically significant positive correlation between LSV2 and weakening of the colonies. Although this result is in agreement with a recent study showing an association between Colony Collapse Disorder (CCD) and LSV1/LSV2 detection [24] further meaningful longitudinal monitoring studies or data from controlled experimental infections are urgently needed to evaluate the role of LSVs in both individual bee and bee colony health.

Although chronic bee paralysis virus (CBPV) is long since known and its pathology as well as transmission routes are well described [25,26,27,28], molecular data on this virus going beyond genomic data [29,30,31] are scarce. Consequently, the study “Characterisation of Structural Proteins from Chronic Bee Paralysis Virus (CBPV) Using Mass Spectrometry” by Chevin and co-workers [32] focused on sequencing virion proteins of CBPV in order to identify (i) the number of proteins involved in capsid formation and (ii) the RNA and the genes encoding these proteins. CBPV is a single-stranded RNA virus. Its genome comprises two major RNAs, RNA1 and RNA2 [33]. It was suggested that the structural proteins of CBPV are encoded by ORF2 and ORF3 located on RNA2 [31]. The data presented here confirmed that CBPV virions contain two proteins encoded by ORF2 and ORF3 of RNA2. In addition, based on the obtained peptide sequence data, a third virion protein translated from RNA2 was postulated although the underlying translation mechanism remained elusive.

Members of the ABPV/KBV/IAPV (acute bee paralysis virus/Kashmir bee virus/Israeli acute bee paralysis virus) group of bee pathogenic viruses are consistently implicated in colony losses in many regions of the world [34,35,36,37,38,39]. Therefore, several attempts have been made to develop antiviral treatment strategies in honey bees via the use of double stranded RNA (dsRNA) to exploit the RNA interference pathway of the insect’s innate immunity [40,41,42,43]. IAPV was originally identified as a bee-specific virus [44] but it is now known to have a much broader host spectrum including wild social and solitary bees as well as social wasps [45,46]. Considering the importance of wild pollinators for human food security [47] and their menace through pathogen spillover from managed honey bee colonies [48] studies on viral infections of bumblebees and their possible treatments are timely and needed. In their study “The Effect of Oral Administration of dsRNA on Viral Replication and Mortality in *Bombus terrestris*”, Piot and co-workers [49] performed experimental IAPV infection of bumblebees and investigated viral pathology, tissue tropism, and virulence. The authors then orally administered IAPV-specific dsRNAs in order to reduce viral titers in infected bumble bees. Surprisingly, the non-specific control dsRNA, which was based on the GFP nucleotide sequence, was more effective than the IAPV-specific dsRNA. The relative normalized IAPV titer in heads, guts, and fat bodies of infected individuals was significantly decreased with the negative control dsRNA, i.e., a dsRNA specific for a region of the gene coding for green fluorescent protein (dsGFP), compared to the dsRNA which was virus-specific. Likewise, mortality of IAPV infected bumblebees was significantly reduced with the non-specific dsGFP whereas the IAPV-specific dsRNA had little effect. These findings are consistent with a recent similar finding in honey bees [50] suggesting that at least in bees (Apidae) or maybe even in Hymenoptera, any dsRNA able to enter cells can serve as a pathogen associated molecular pattern (PAMP) and trigger the intracellular anti-viral immune response.

## 3. General Conclusion

This special issue on honey bee viruses demonstrates impressively how diverse this field is. There still exist many unknown honey bee pathogenic viruses which await discovery, either occasionally or more target-oriented with sequencing approaches, and subsequently analyzed. Furthermore, the existing knowledge on the well-characterized honey bee viruses has to be considerably extended. Therefore, much work is still to be done to get a comprehensive understanding of the virology of the honey bee.
